# Supplementary material for: Identifying core leadership competencies to success non-communicable disease control and prevention programs: A mixed-methods study
Source: PLoS One. 2025 Apr 9;20(4):e0320707. doi: 10.1371/journal.pone.0320707 (PMC12007618; doi:10.1371/journal.pone.0320707)
Supplement: S1 Checklist — (DOCX) [file pone.0320707.s001.docx]

**Consolidated criteria for reporting qualitative studies (COREQ): 32-item checklist**

Domain 1: Research team and reflexivity

Personal Characteristics

1. Interviewer/facilitator

Which author/s conducted the interview or focus group? **Yegane Partovi**, **Jafar sadegh Tabrizi**

2. Credentials

What were the researcher’s credentials? **PhD**

3. Occupation

What was their occupation at the time of the study? **Assistant professor, professor**

4. Gender

Was the researcher male or female? **Female and** male

5. Experience and training

What experience or training did the researcher have? **Various Research workshop (qualitative and quantitative) and published articles**

Relationship with participants

6. Relationship established

Was a relationship established prior to study commencement? **At the start of each discussion, by explaining the purpose of the study and ensuring the confidentiality of the content of the interviews and anonymity, discussions were taped with 2 sound recorders.**

7. Participant knowledge of the interviewer

What did the participants know about the researcher? **Participants knew reasons for doing the research and the purpose of the study**

8. Interviewer characteristics

What characteristics were reported about the interviewer/facilitator? **N/A** or **None**

Domain 2: study design

qualitative

10. Sampling

How were participants selected? **Purposive**

11. Method of approach

How were participants approached? **Face-to-face and meeting**

12. Sample size

How many participants were in the study? **10**

13. Non-participation

How many people refused to participate or dropped out? Reasons? **None**

Setting

14. Setting of data collection

Where was the data collected? **Workplace or everywhere they wanted**.

15. Presence of non-participants

Was anyone else present besides the participants and researchers? **NO, None**

16. Description of sample

What are the important characteristics of the sample? **The inclusion criteria consisted of having at least three years of management experience or executive and scientific activities in the field of PHC and NCDs and having the qualifications and willingness to attend the interviews.**

Data collection

17. Interview guide

Were questions, prompts, guides provided by the authors? Was it pilot tested? **Yes. It was.**

18. Repeat interviews

Were repeat interviews carried out? If yes, how many? **Yes. They were. We have two in-person expert panels (for 60 minutes each time)**

19. Audio/visual recording

Did the research use audio or visual recording to collect the data? **Audio recording**

20. Field notes

Were field notes made during and/or after the interview or focus group? **Researcher take small, key word base notes based on during the discussion, while maintaining participation and eye contact with participants.**

21. Duration

What was the duration of the interviews or focus group? **60 minutes**

22. Data saturation

Was data saturation discussed? **Yes**

23. Transcripts returned

Were transcripts returned to participants for comment and/or correction? **Yes**

Domain 3: analysis and findingsz

Data analysis

24. Number of data coders

How many data coders coded the data? **2**

25. Description of the coding tree

Did authors provide a description of the coding tree? **N/A**

26. Derivation of themes

Were themes identified in advance or derived from the data? **No, they derived from the data**

27. Software

What software, if applicable, was used to manage the data? **N/A**

28. Participant checking

Did participants provide feedback on the findings? **Yes**

Reporting

29. Quotations presented

Were participant quotations presented to illustrate the themes / findings? Was each quotation identified? **Yes**

30. Data and findings consistent

Was there consistency between the data presented and the findings? **Yes**

31. Clarity of major themes

Were major themes clearly presented in the findings? **Yes, they were presented**

32. Clarity of minor themes

Is there a description of diverse cases or discussion of minor themes? **Yes, they were presented**.
