# Supplementary material for: Identifying core leadership competencies to success non-communicable disease control and prevention programs: A mixed-methods study
Source: PLoS One. 2025 Apr 9;20(4):e0320707. doi: 10.1371/journal.pone.0320707 (PMC12007618; doi:10.1371/journal.pone.0320707)
Supplement: Attachment — Submitted filename: Response to Reviewers 3.docx [file pone.0320707.s005.docx]

“Reviewer #1: Recommended corrections are well done, all corrections have been addressed. I thank the authors for making these corrections. Thank you.

Reviewer #2: Dear Authors, dear Editor,

the comments I have made in the frist revision have been largely adressed. However there are still points that I have to note, some of them for the third time:

1. Language. I have made some suggestions previously, but suggested proofread by the authors. It appears that there was no effort to go beyond the minimum reuirements of my comments. Errors persist such as competences/competencies; NCD not introduced in introduction, new limitation section overall not fluent; Table 1 mining spaces, title misspelled, cursive; Figure 1 in bad quality (perhaps due to upload); reports excluded not in uniform fashion, use of "=", " =", " = "; Appendix named Delphii. In the way the manuscript is presented I do not feel that the language concerns are taken serious in any way.

I hope this message finds you well. As we welcome the New Year, I wanted to take a moment to express my heartfelt gratitude for your valuable contributions as a reviewer. Your insights and feedback have been instrumental in enhancing the quality of our work. Wishing you a joyful and prosperous New Year filled with success and happiness. May 2025 bring you new opportunities and continued achievements.

The Prisma is written according to the provided Prisma 2020 template (<https://www.prisma-statement.org/prisma-2020-flow-diagram>).

The text of the manuscript was reviewed by a linguistic editor and changes were made. The changes are highlighted in turquoise color.

2. Methodology 150 to 19. You state that
a) Based on the expert panel input, the competencies were categorizing into four domains titled specific, distinct, managerial and behavioral.
b) Based on the Delphi results, the competencies were categorized into four domains titled specific, distinct, managerial and behavioral
Apart from the error of "categorizing", it is yet to be explaied where the 19 domains come from (based on the 150) or where the difference of these sentences lies. The grouping of domains and categorizing are (in my understanding) two different steps. Figure 2 is unfortunately of no help in understanding the process. I recommend to delete the figure and replace it by a) a better flow of competencies or b) extended explanation in text.

Thank you for your valuable feedback and for highlighting areas (turquoise color) in need of further clarification. We have revised the manuscript to better explain the process of categorizing competencies from the initial 150 to the final 15, as well as the development of the four overarching domains: specific, distinct, managerial, and behavioral. Below, we address the specific points you raised:

1. **Clarification of Process Steps**: We have provided an expanded explanation in the methodology section to clarify the process of refining and categorizing the competencies:
   - **Initial Extraction and Reduction**: After identifying 150 competencies, an initial review was conducted by the expert panel. Each panel member independently analyzed the competencies and suggested reductions and merges, which were then compiled and presented in a series of meetings for further refinement. This iterative review process helped us reduce the list to a manageable set of competencies.
   - **Categorization Framework Development**: Following the reduction process, we identified the need for a structured categorization framework. After reviewing several competency models, we chose the Bartram 8G Model, which encompasses competency domains such as Analysis & Interpretation, Interaction & Influence, Support & Cooperation, Decision Making, Action & Execution, Adaptation & Adjustment, Organization, and Creation & Conceptualization. This model provided a solid foundation for organizing our competencies.
   - **Final Categorization and Customization**: We consolidated similar competencies within the 8G framework and refined them into four key domains: specific, distinct, managerial, and behavioral. Each domain was defined based on the thematic commonalities among competencies. These final categorizations were reviewed and endorsed by the expert panel in a subsequent session.
2. **Revision of Figure 2**: We have updated Figure 2 to present a clearer step-by-step flowchart illustrating the categorization process, showing how the competencies were refined from 150 to 15 and organized into four domains.

We appreciate your guidance, which has helped us enhance the clarity and flow of our methodology. Please let us know if further clarification is needed.
