# Supplementary material for: Identifying core leadership competencies to success non-communicable disease control and prevention programs: A mixed-methods study
Source: PLoS One. 2025 Apr 9;20(4):e0320707. doi: 10.1371/journal.pone.0320707 (PMC12007618; doi:10.1371/journal.pone.0320707)
Supplement: Attachment — Submitted filename: Response to Reviewers.docx [file pone.0320707.s003.docx]

**Point-by-point response to reviewers**

Dear Editor- in- Chief

Our thanks to the reviewers and the subject editor for thoughtful critiques of our manuscript. We have adopted all of the suggestions. We think that the manuscript has been greatly improved by these revisions and we hope that you will now find it suitable for publication in PLOSONE. Our point-by-point responses to comments are detailed on the following pages.

Best regards,
Yegane Partovi
Corresponding Author

Response to academic editor:

In your Data Availability statement, you have not specified where the minimal data set underlying the results described in your manuscript can be found. PLOS defines a study's minimal data set as the underlying data used to reach the conclusions drawn in the manuscript and any additional data required to replicate the reported study findings in their entirety. All PLOS journals require that the minimal data set be made fully available. For more information about our data policy, please see <http://journals.plos.org/plosone/s/data-availability>. All of attachment was provided in supplementary file1.

. Please include your full ethics statement in the ‘Methods’ section of your manuscript file. In your statement, please include the full name of the IRB or ethics committee who approved or waived your study, as well as whether or not you obtained informed written or verbal consent. If consent was waived for your study, please include this information in your statement as well. full ethics statement in the ‘Methods’ section was added.

Reviewer #1: Abstract

1. It seems that in this study, "Core Leadership Competencies to Success Non-Communicable Disease Control and Prevention Programs", have only been identified; And the word "...Developing" is beyond and wider than what is done in this study. If the authors' team deems it appropriate, I suggest that a more appropriate word be substituted for the term "Develop*" in the entire article (title, abstract and main text). The word 'identify' has been substituted for the term 'develop' in the entire article (title, abstract and main text). Amendments are indicated in the text by yellow highlighting.
2. In the results section of the abstract, "inspiration" is written as one of the main competencies, while it is not mentioned in Table 2, which lists the competencies. Please be consistent about it throughout the article. Amendments have been made. They are indicated in the text by yellow highlighting.
3. Please use a specific term, and not its synonyms, in introducing and talking about leadership competencies throughout the article. For example, although you have used the term "planning" in the abstract, you have used the term "Plan Making" in other parts of the article. Please maintain consistency. Amendments have been made. They are indicated in the text by yellow highlighting.

4. In writing the purpose of the article (This study developed leadership competencies for managers of non-communicable diseases in Iran.), I suggest that it be clearly stated that the purpose of the study is to identify these competencies. Amendments have been made. They are indicated in the text by yellow highlighting.

Introduction
1. Please correct the following sentence by writing more up-to-date statistics: "According to statistics from 2019, NCDs were the cause of 74.4% of all deaths and 63.8% of years lived with a disability (1).” The latest statistics provided in 2019 are cited in the article text according to the research conducted by this study's researchers. All the reliable and relevant sources in this field including WHO, WORLD BANK….. were examined. The latest statistics are for 2019.
2. In connecting the two main issues of the study (i.e., "non-communicable diseases" and "leadership competencies"), there is a lot of content weakness. The authors have used the following phrase to connect these two issues, while it has no reference and does not seem very appropriate.
(These capacities are essential prerequisites to implement leadership skills who can coordinate, support, and ensure their long-term continuity in order to produce beneficial outcomes. Leaders with required competencies and qualifications is important as essential to ensure effective interventions are delivered as efficiently as possible within the available resources, especially in low- and middle-income countries.)
Please use specific scientific references in creating a connection between these two main issues, and connect them with a literature justifying them. Amendments have been made. They are indicated in the text by yellow highlighting.
3. Please rewrite and correct the following parts by referring to scientific references.
(In order to develop leadership and management expertise in those who will be responsible for the implementation and running of NCD prevention and control programs, it is essential to identify the required competencies. Globally, existing educational programs for primary health care (PHC) managers lack clear leadership and management competencies for PCNCD program5 management.) Amendments have been made. They are indicated in the text by yellow highlighting.

4. Please provide evidence of the failure of specific health programs in Iran and the world, that the cause of the failure was actually ineffective management. The main purpose of this study is not to investigate managerial inefficiency and the failure of health programs due to the inefficiency of managers. The current education programs for PHC managers worldwide do not provide clear leadership competencies for NCD program management.

***

Methods
1. Inclusion and exclusion criteria related to literature review should be written. Amendments have been made. They are indicated in the text by yellow highlighting.


***

Results
1. In writing the title of Table 3, the word "remaining" is not necessary. Amendments have been made.
2. The explanations related to Table 3 should be written above it. Amendments have been made.
. They are indicated in the text by yellow highlighting.
***

Conclusion
1. The following text needs a reference. (Planning, risk, and disaster management skills were determined to be the most crucial competencies, according to the literature review.) Some references have been added. They are indicated in the text by yellow highlighting.
2. Please remove the sentences without reference (except in permitted cases) from the text of the manuscript or rewrite them based on a valid reference (for example: Consequently, to achieve the best outcomes of NCDs programs with available resources requires managers accepting of and skilled in teamwork and the ability for using collective wisdom.) Some references have been added. They are indicated in the text by yellow highlighting.

3. In general, the discussion section does not meet my expectations of a good discussion. Please discuss each of the four areas ('specific', 'distinct', 'managerial' and 'behavioral') in 4 separate paragraphs, write about the leadership competencies in each area and their supporting evidence. Write more clearly about the area under discussion and the competencies covered by it.
4. Strengths and limitations of the study are not mentioned. Amendments have been made.
. They are indicated in the text by yellow highlighting.

Reviewer #2: Dear Authors,

I have concluded my review with minor revisions to share. The introduction offers a fond overview to the relevance of NCDs and their health-economic implications. I have no further remarks to the motivation and discussion.
Following remarks should be considered for the methodology:
- Was an appropriate study guideline followed? If so, please indicate in the text or carry out this step. One of the possibilities should be the SQUIRE Guideline. Amendments have been made.
. They are indicated in the text by green highlighting.

- Ll.149 According to the PLOSONE Guidelines the identified studies should be shared in order to provide a transparent methodology. Also please report the search strategy and consider the use of a flow diagram and explain the concatenation of the search strings (see below) Amendments have been made. The identified studies provided in Attachment file 1.
- Ll.149 Please explain the revision of competencies more precisely, respectively why there was no change in the absolute numbers. Amendments have been made. They provided in table1.
- L.173 The source on the DELPHI panel procedure is unclear. Please cite in the text wat (modified) methodology was used. Some references have been added. They are indicated in the text by green highlighting.

- L.177 sentence structure not straight forward. . Amendments have been made.
- L.195 Please explain precisely how many rounds were made if not two, respectively how the final decision was made. Amendments have been made. They are indicated in the text by green highlighting.

The 19 leadership competencies entered the first round of the Delphi phase with near 90% of invited participants responding. The titles of 4 domains were agreed upon by all participants. The ‘risk management’ competency was merged with the ‘disaster management’ competency and the competencies of ‘resource, executive and administrative management’ were merged as ‘planning’. The ‘managing change’ competency was rephrased into ‘leading and managing change’. Competencies that received 80 to 100% agreement in the first round of the Delphi process were accepted without further testing, while those that received lower than 80% agreement underwent revision based on participant comments. Four domains and fifteen leadership competencies were presented for the second round of Delphi after modification and editing. Again, there was no disagreement on the four fields. We concluded that saturation and consensus had been reached because no additional competencies were proposed in the second round.
- L.217 Where does the “19” come from? Amendments have been made. They provided in table1.
- L. 241 Having introduced the first set of competencies is a bold assumption, especially where the overall topic is not restricted to Iran. Please state explicitly what new findings the study brings.

Globally, a few educational programs have been created and put into place to improve the leadership and management competencies of primary health care managers, but none of them have specifically addressed the leadership competencies needed for managers of NCDs. As a result, our study developed leadership competencies for managers of non-communicable diseases in Iran for the first time.
- Table 3: Give the competencies ranked by importance. The objective of this study was to identify the leadership competences required for managers of non-communicable diseases. Prioritizing the competencies identified in the present study using the AHP method is possible, but it wasn't one of our objectives.
- Table 4: Adjust the formatting uniformly, especially the use of “-“ Amendments have been made.
