# Supplementary material for: Identifying core leadership competencies to success non-communicable disease control and prevention programs: A mixed-methods study
Source: PLoS One. 2025 Apr 9;20(4):e0320707. doi: 10.1371/journal.pone.0320707 (PMC12007618; doi:10.1371/journal.pone.0320707)
Supplement: Attachment — Submitted filename: Response to Reviewers2.docx [file pone.0320707.s004.docx]

**Point-by-point response to reviewers**

Dear Editor- in- Chief

Our thanks to the reviewers and the subject editor for thoughtful critiques of our manuscript. We have adopted all of the suggestions. We think that the manuscript has been greatly improved by these revisions and we hope that you will now find it suitable for publication in PLOSONE. Our point-by-point responses to comments are detailed on the following pages.

Best regards,
Yegane Partovi
Corresponding Author

Reviewer #1:

We appreciate the valuable feedback from you, which has significantly improved our work.

1. In lines 70 and 72, in two consecutive sentences, the word ""According to"" is repeated; Please avoid repetition. Amendments have been made. They are indicated in the text by gray highlighting.

2. Although you have added a sentence about the strengths and weaknesses of the research, it needs further elaboration. Please write more about the strengths and weaknesses of the research. The strengths and weaknesses of the research have been added. They are indicated in the text by gray highlighting.

Reviewer #2:

Thank you for your insightful comment. We appreciate the valuable feedback from you, which has significantly improved our work. We try to provide the valid response based on our knowledge. We would be grateful if you could kindly accept our responses.

Overall statement: Many of my previous comments were addressed, though not all. I have marked some additional problems in language, but certainly a precise reread of the authors is necessary to eliminate formatting and language errors, otherwise it does not seem like the critics were taken seriously. Moreover, the methodological description lacks the necessary clarity, see comments below. I strongly recommend the use of clearer flowcharts for 1. Literature process and 2. Competencies process. Amendments have been made. Fig 1 and fig 2 have been added. They are indicated in the text by purple highlighting.

Introduction:
Please use primary sources when citing counts and costs of NCD. In particular, the way reference (2) is used is not valid. Amendments have been made. They are indicated in the text by purple highlighting.
The statements made about NCD should be more conditional unless proven upon no doubt.

Methods:
The filled in COREQ checklist should be provided in appendix. It was provided.
Again consider the use of a flow diagram. How many studies were duplicates, how many studies were screened? Were the keywords used separately or with OR/AND concatenation? What were the main results of exclusion? Amendments have been made. Fig 1 and table 1 (the search strategy) have been added in method section. They are indicated in the text by purple highlighting.

Table 1 is more a figure that should be focussing more on visualizing the flow from 150 to 15. Duplicates merge etc. should be reported clearly. Fig 2 has been included. They are indicated in the text by purple highlighting.

State the reason for dividing expert and Delphi round and the main difference. Was consensus in the DELPHI panel assessed? Why? Two in-person expert panels (for 60 minutes each time) were held to evaluate, screening and make changes to the initial draft leadership competencies. consensus was assessed in the DELPHI panel. Amendments have been made. You can see them in expert panel and Delphi technique survey sections. They are indicated in the text by purple highlighting.
Was an 80% response rate reached? The consensus was reached when 80% of the participants rated a competency above 7 in each round. The response rate of the participants near 90%. Were do you explain the consensus from table 1? we explain it in Delphi technique survey sections. Competencies that received 80 to 100% agreement in the first round of the Delphi process were accepted without further testing, while those that received lower than 80% agreement underwent revision based on participant comments. They are indicated in the text by purple highlighting.
Results:
Consider including all 15 competencies in table 3 for clarity. How is table 3 ordered? Consider stating by domains and alphabetically. We appreciate this suggestion. Table 4 and table 5 have been modified by alphabetically. They are indicated in the text by purple highlighting.
Are the included papers cited in the appendix? 15 references have been added.

l.71 insert space before (1). They are indicated in the text by purple highlighting.
ll.82 Make clear (with source) that the UN MDG is in danger, the threatening spread is a logical consequence. They are indicated in the text by purple highlighting.
l. 80 insert space. They are indicated in the text by purple highlighting.
l. 93 formatting of citation. They are indicated in the text by purple highlighting.
l. 112 formatting. They are indicated in the text by purple highlighting.
l. 113 as far as known to the authors? They are indicated in the text by purple highlighting.
l. 118 remove space, insert space They are indicated in the text by purple highlighting.
l. 120 between … and They are indicated in the text by purple highlighting.
l. 135 of the Tabriz? They are indicated in the text by purple highlighting.
l. 136 remove space They are indicated in the text by purple highlighting.
l. 147 or English They are indicated in the text by purple highlighting.
l. 184 language They are indicated in the text by purple highlighting.
l. 193 language They are indicated in the text by purple highlighting.
l. 234 Management ◊ Resource, executive and administrative management? They are indicated in the text by purple highlighting.
